# The Late Embryogenesis Abundant Proteins in Soybean: Identification, Expression Analysis, and the Roles of GmLEA4_19 in Drought Stress

**DOI:** 10.3390/ijms241914834

**Published:** 2023-10-02

**Authors:** Binhui Guo, Jianhua Zhang, Chunhong Yang, Lu Dong, Heng Ye, Babu Valliyodan, Henry T. Nguyen, Li Song

**Affiliations:** 1 Joint International Research Laboratory of Agriculture and Agri-Product Safety, The Ministry of Education of China, Yangzhou University, Yangzhou 225009, China; bhguo@yzu.edu.cn (B.G.); zjh0007@foxmail.com (J.Z.); azneych@outlook.com (C.Y.); dl0828@foxmail.com (L.D.); 2 Zhongshan Biological Breeding Laboratory, No. 50 Zhongling Street, Nanjing 210014, China; 3 Division of Plant Sciences, University of Missouri, Columbia, MO 65211, USA; yehe@missouri.edu (H.Y.); nguyenhenry@missouri.edu (H.T.N.); 4 Department of Agriculture and Environmental Sciences, Lincoln University, Jefferson City, MO 65101, USA; valliyodanb@lincolnu.edu

**Keywords:** *LEA* gene family, soybean, genome-wide identification, evolutionary analysis, abiotic stress, *GmLEA4_19*, drought tolerance

## Abstract

Late embryogenesis abundant (LEA) proteins play important roles in regulating plant growth and responses to various abiotic stresses. In this research, a genome-wide survey was conducted to recognize the LEA genes in *Glycine max*. A total of 74 GmLEA was identified and classified into nine subfamilies based on their conserved domains and the phylogenetic analysis. Subcellular localization, the duplication of genes, gene structure, the conserved motif, and the prediction of cis-regulatory elements and tissue expression pattern were then conducted to characterize GmLEAs. The expression profile analysis indicated that the expression of several GmLEAs was a response to drought and salt stress. The co-expression-based gene network analysis suggested that soybean LEA proteins may exert regulatory effects through the metabolic pathways. We further explored GnLEA4_19 function in *Arabidopsis* and the results suggests that overexpressed GmLEA4_19 in *Arabidopsis* increased plant height under mild or serious drought stress. Moreover, the overexpressed GmLEA4_19 soybean also showed a drought tolerance phenotype. These results indicated that GmLEA4_19 plays an important role in the tolerance to drought and will contribute to the development of the soybean transgenic with enhanced drought tolerance and better yield. Taken together, this study provided insight for better understanding the biological roles of *LEA* genes in soybean.

## 1. Introduction

The Late Embryogenesis Abundant (LEA) protein family is a large group of proteins that accumulate during the late stages of seed development or in vegetative tissues in response to environmental stresses such as drought, salinity, and cold, as well as the exogenous application of abscisic acid [1]. LEA proteins are hydrophilic, rich in glycine, and have a low molecular weight (10–30 kDa). These proteins have been shown to protect plant metabolism against abiotic stresses with properties that include antioxidant activity, scavenging active oxygen free radicals, metal ion binding, membrane and protein stabilization, hydration buffering, and DNA and RNA interactions. They play a crucial role in equipping seeds to survive by maintaining minimal hydration levels in the dry organism and preventing the denaturation of cytoplasmic components [2,3]. In higher plants, *LEA* gene families have been identified and analyzed at the whole-genome level in several sequenced plant species, such as *Arabidopsis* [4,5], rice [6], maize [7], *Brassica napus* [8], upland cotton [9], sorghum [10], and wheat [11]. 

Previous studies have shown that LEA proteins play a key role in plant resistance to drought, salt, heat, and cold. For example, the overexpression of *AtLEA3-3* in *Arabidopsis* promotes vegetative growth and enhances water retention ability [12]. Similarly, the overexpression of *OsLEA3-1* and *OsLEA3-2* in transgenic rice plants enhances its tolerance to drought [13,14]. The overexpression of the wheat *LEA3* gene (WZY3-1) in *Arabidopsis* also enhances their tolerance to drought [15]. In another study, the overexpression of *TaLEA3* in *P*. *amurense* improved its drought resistance by promoting the rapid stomatal closure under drought stress conditions [16]. The overexpression of the pepper dehydrin gene *CaDHN5* in transgenic Arabidopsis also showed enhanced tolerance to salt and osmotic stresses [17]. The overexpression of the *ZmDHN15* gene has been shown to effectively improve cold stress tolerance in both yeast and Arabidopsis [18]. Additionally, the overexpression of *MsLEA4-4* in *Arabidopsis* conferred late-germination phenotypes and a higher survival rate compared to WT plants under salt stress and abscisic acid treatment [19]. Finally, Lv et al. found that the overexpression of *MsLEA-D34* in *Arabidopsis* causes increased tolerance to osmotic and salt stresses and resulted in an early flowering phenotype under drought or well-watered conditions [20]. The overexpression of *LEA3* gene (Gh_A08G0694) significantly enhances drought and salinity stress tolerance in transgenic cotton [21]. Overall, these studies demonstrate the potential of LEA proteins as a tool for improving plant stress tolerance. 

Soybean is a major source of the vegetable protein and edible oil, but its production is threatened by various abiotic stresses such as drought, salinity, and osmotic stress [22]. It was reported that GmLEA4 (GmPM1 and GmPM9) and GmASR proteins can combine with metal iron such as Fe^3+^, Ni^2+^, Cu^2+^, and Zn^2+^, which may help protect cells by reducing the toxicity of these ions [23,24]. The overexpression of *GmLEA2-1* in transgenic *Arabidopsis* has been shown to increase tolerance to drought and salt stress [25]. The GmDHN1 protein has a very low intrinsic ability to adopt an α-helical structure and interact with phospholipid bilayers through amphipathic α-helices, which enables it to remain in a highly extended conformation at low temperatures and play an important role in preventing freezing, desiccation, ionic, or osmotic stress-related damage to macromolecular structures [26]. 

It can be predicted that further research on the function and expression regulation of GmLEA proteins could provide a more comprehensive understanding of the physiological and biochemical mechanisms of plant response to drought. This could certainly provide a theoretical basis for the development of new drought-resistant varieties. In this study, a genome-wide identification of *LEA* genes in the *Glycine max* genome was performed. Furthermore, the gene structure, protein motif composition, chromosome location, cis-acting elements of genes, recombination events, selective stress, functional networks, and expression profiles under drought and salt treatments were investigated. Additionally, the overexpression of *GmLEA4_19* in *Arabidopsis* and soybean was developed and the transgenic plants showed an enhanced drought tolerance phenotype. These results provide a theoretical basis for the molecular evolution and functional research of the *GmLEA* gene family in soybean.

## 2. Results

### 2.1. Identification and Characterization of the LEA Genes in Glycine Max

By combining local BLAST and HMM methods, a total of 74 *LEA* genes were identified in the genome of *G. max*. According to sequence homology and conserved motifs in the Pfam database, these *GmLEA* genes were classified into nine subfamilies, namely GmLEA1-6, dehydrin (DHN), ASR, and SMP (Table 1). In addition to the DHN and SMP subfamilies, the soybean genome contained more genes in other subfamilies than the *Arabidopsis* genome, especially in the LEA_3 and LEA_4 subfamilies. The LEA4 subfamily was the largest, with 27 members (Table 1). The GmASR subfamily was found exclusively in the soybean genome, while the EM subfamily was absent.

**Figure 1:**
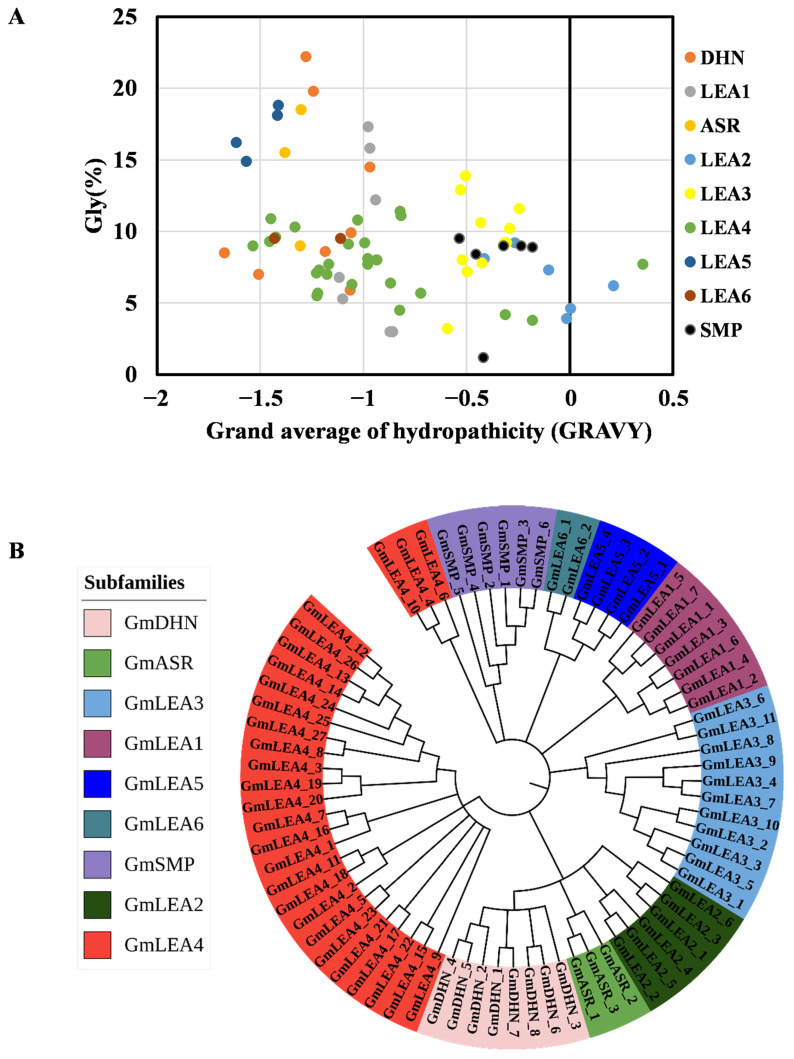
Characterization of proteins and phylogenetic evolutionary relationship of GmLEA proteins. (**A**) Plot of grand average of hydropathicity (GRAVY) and glycine content (%) in each GmLEA protein. (**B**) Phylogenetic evolutionary relationship of GmLEAs. The phylogenetic tree was constructed from 74 GmLEA members with the neighbor-joining method using MEGA 11.

The physicochemical parameters of each GmLEA protein were calculated using ExPASy. The GmLEA4, GmSMP, and GmDHN subfamilies contained a greater number of amino acid residues than other LEAs. Members of the GmLEA3 subfamily all have low molecular masses (Table 1). Half of GmLEA proteins have relatively low isoelectric points (pI < 7), including the GmLEA2, GmLEA5, GmASR, and GmSMP subfamilies. The pI values of remaining proteins were greater than 7, particularly in the GmLEA1 and GmLEA3 subfamilies (Table 1). The grand average of the hydropathy index (GRAVY) was defined using the sum of the hydropathy values of all amino acids divided by the protein sequence length and was used to represent the hydrophobicity value of a peptide. Positive GRAVY values represent hydrophobicity and negative values indicate hydrophilicity. The GRAVY value of most GmLEA proteins was less than 0, suggesting that a large proportion of the GmLEA proteins were hydrophilic. GmLEA2_2, GmLEA2_5, and GmLEA4_6 were hydrophobic proteins with a GRAVY value more than 0. In addition, most of the GmLEA proteins contained over 5% glycine (Figure 1A). 

The prediction of the subcellular location showed that nearly 80% of GmLEA proteins were present in the nucleus. Only three GmLEA proteins (GmLEA2_4, GmLEA4_6, and GmSMP_4) were predicted to have a high possibility of being in the cell membrane. Interestingly, seven GmLEA3 proteins may be found in the chloroplast base on Plant-mPLoc software prediction, where most of these members were also predicted to be found in the mitochondrion based on the PProwler software prediction. Moreover, several GmLEA4 proteins were predicted to be in the cell wall and four GmDHN proteins may be distributed in the cytoplasm. Six members of the GmLEA4 subfamily are predicted to participate in the secretory pathway (Table S1).

### 2.2. Phylogenetic Tree, Gene Structure, and Conserved Motifs Analysis of GmLEA Genes

A phylogenetic tree was constructed using the neighbor-joining (NJ) method to analyze all GmLEAs, as shown in Figure 1B. The proteins were clustered into nine groups, in which the LEA4 group was divided into two sub-clusters. Clusters GmLEA2, GmASR, and GmDHN were part of a larger clade, while GmLEA5 and GmLEA6 were also grouped into a larger clade. 

**Figure 2:**
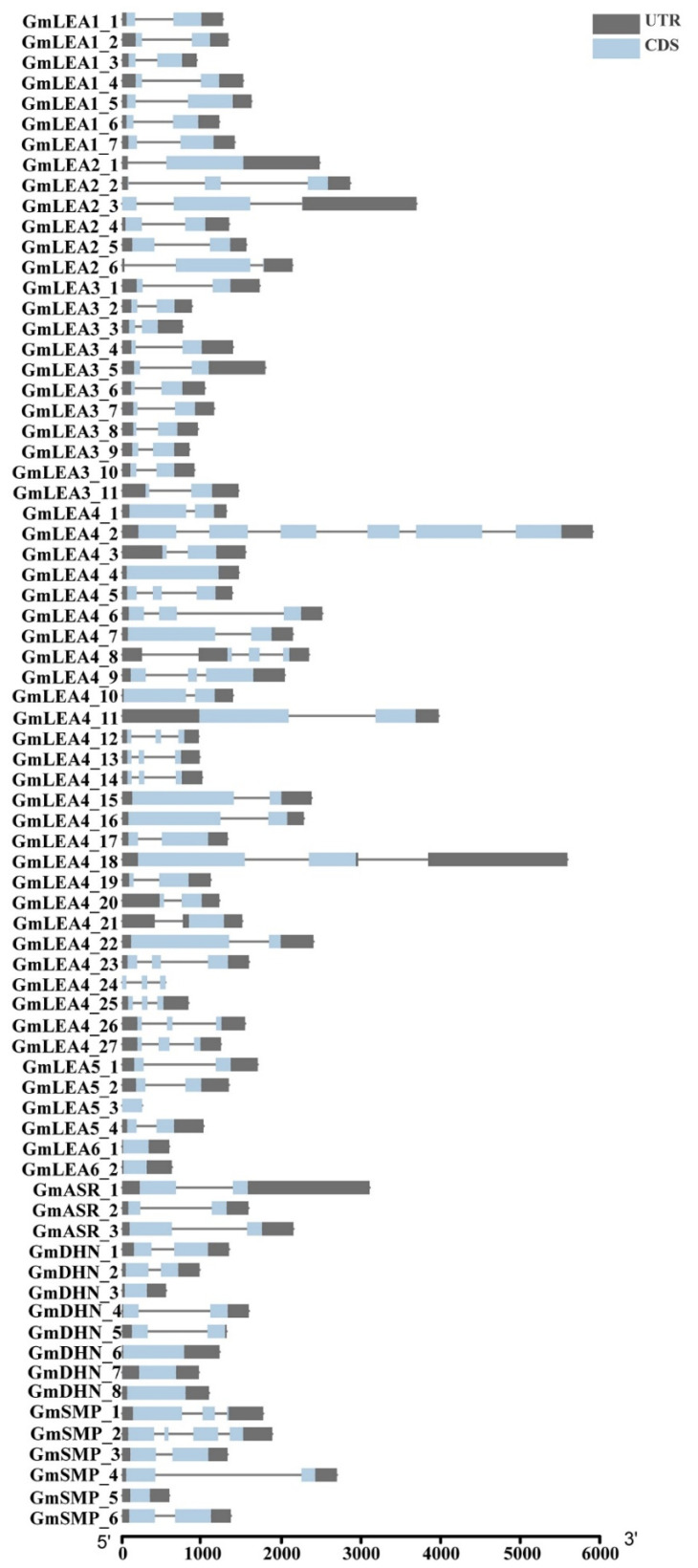
Gene structures of GmLEAs. The blue boxes represented exons and the black line represented intron. The gray boxes represented the UTRs. The scale at the bottom showed the exon sizes.

The exon–intron organization analysis was performed to characterize the structural diversity of GmLEA proteins. Most *GmLEA* genes contain one to three exons, except for *GmLEA4_2* and *GmSMP2*, which have six and four exons, respectively. There were eight genes in this gene family with only one exon, such as *GmDHN_3*, *GmDHN_6*, *GmDHN_7*, *GmDHN_8*, *GmSMP_5*, *GmLEA4_4*, *GmLEA6_1*, and *GmLEA6_2* (Figure 2).

**Figure 3:**
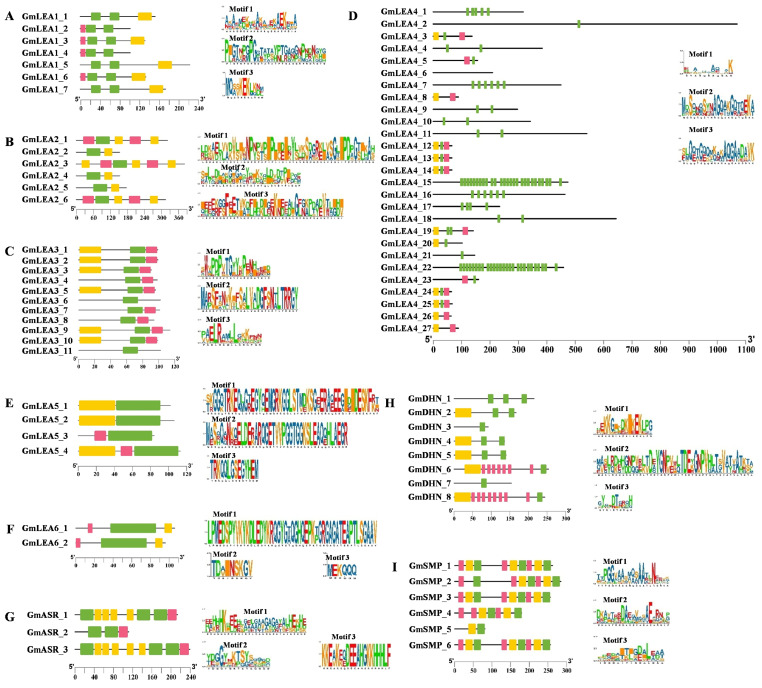
Conserved motif patterns of GmLEAs. Conserved motifs were identified using MEME tools. Three predicted motifs were represented by distinct colored boxes and the grey lines indicated non-conserved regions. (**A**) GmLEA_1 subfamily; (**B**) GmLEA_2 subfamily; (**C**) GmLEA_3 subfamily; (**D**) GmLEA_4 subfamily; (**E**) GmLEA_5 subfamily; (**F**) GmLEA_6 subfamily; (**G**) GmSMP subfamily; (**H**) GmASR subfamily; and (**I**) GmDHN subfamily. Green box: Motif 1; Yellow box: Motif 2; Red box: Motif 3.

Three motifs were identified as conserved motifs in each subfamily. Most of the closely related genes in each subfamily exhibit similar motif compositions, suggesting functional similarities in the LEA subfamily. In the GmLEA1 subfamily, motif 1 repeated two times in each gene. Moreover, motif 1 repeated 27 times in GmLEA4_15 and GmLEA4_22. No conserved motifs were contained in GmLEA4_6. In addition, motifs 1, 2, and 3 form a group and exist in the form of a tandem repeat in the GmSMP subfamily. These results imply that the composition of the structural motifs varies among different LEA subfamilies but is similar within the subfamilies and also that the motifs encoding the LEA domains are conserved (Figure 3).

### 2.3. Chromosomal Distribution, Collinearity, and Ka/Ks Values of GmLEA Family Members

**Figure 4:**
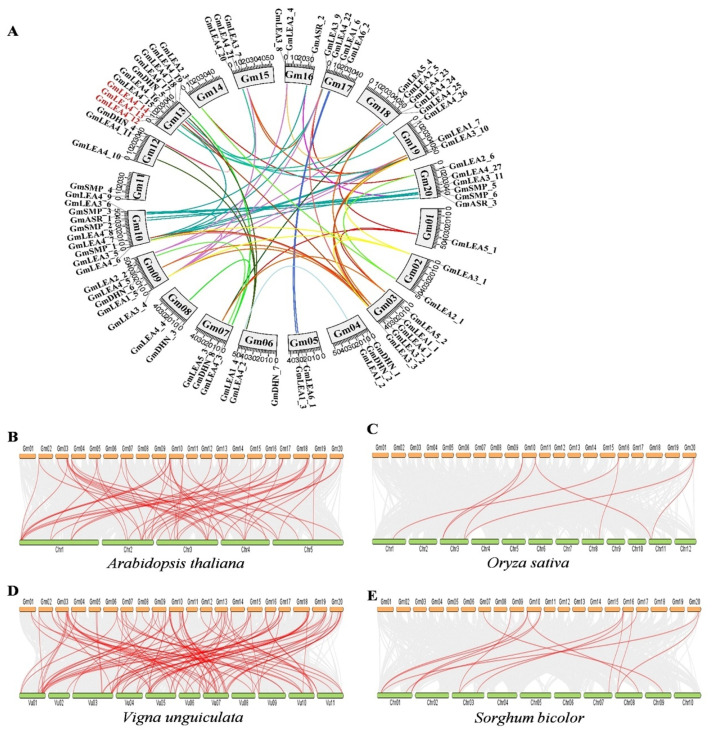
Genomic distribution and synteny analysis of GmLEA family members. (**A**) Genomic distribution and schematic representations for the interchromosomal relationships of 74 *GmLEA* genes across 20 soybean chromosomes. The scale on the circle is in megabases. The numbers of each chromosome are shown inside the circle. The WGD or segmental duplication genes are connected with a line. The colored lines indicate the collinear gene pairs within intrachromosomal and interchromosomal, respectively. (**B**) *Glycine max* vs. *Arabidopsis thaliana*; (**C**) *Glycine max* vs. *Oryza sativa*; (**D**) *Glycine max* vs. *Vigna unguiculata*; and (**E**) *Glycine max* vs. *Sorghum bicolor*. Each horizontal line represents a chromosome. Gray lines in the background indicate the collinear blocks with *G.max* and other four plant species, while the red lines represent the syntenic *LEA* gene pairs. The number represents the corresponding chromosome name.

The chromosomal distribution of the *GmLEA* genes were analyzed and the results showed that 74 *GmLEA* genes were distributed on the 20 chromosomes of *G. max* (Figure 4A). The greatest distribution of *GmLEA* genes was on chromosomes 10 and 13, with nine *GmLEA* genes each, while chromosomes 1 only contained one *GmLEA* gene. Tandem duplication, segmental duplication, and whole-genome duplication correspond to the gene family expansion. Here, we found 57 gene pairs distributed on diverse chromosomes, suggesting that segmental duplication is the primary expansion model of the soybean *LEA* gene family. The soybean genome underwent two rounds of whole-genome duplication, which occurred 59 and 13 million years ago. The expansion of *GmLEA* genes has arisen more recently due to soybean-specific duplication. On the contrary, several tandemly duplicated genes (*GmLEA2_5* and *GmLEA4_23*; *GmLEA4_24* and *GmLEA4_25*; *GmLEA4_12*, *GmLEA4_13,* and *GmLEA4_14*; *GmDHN_1* and *GmDHN_2*; *GmLEA4_5* and *GmLEA2_2*; and *GmSMP_5* and *GmSMP_6*) located on chromosomes 4, 9, 13, 14, 18, and 20 were identified, indicating that tandem duplication also contributes to the expansion of the GmLEA family (Figure 4A). 

To further explore the evolutionary history of the members of the LEA family in *Glycine max*, we constructed a collinear map of *LEA* gene members in *Glycine max* along with two dicotyledons (*Arabidopsis thaliana* and *Vigna unguiculata*) and two monocotyledons (*Oryza sativa* and *Sorghum bicolor*) (Figure 4B–E). The results showed that there were 60 repetitive events in *Arabidopsis thaliana*, 102 repetitive events in *Vigna unguiculata*, eight repetitive events in *Oryza sativa*, and 14 repetitive events in *Sorghum bicolor*, respectively (Table S2). We found that several GmLEAs, such as GmLEA2_4, GmLEA3_10, GmSMP1, and GmSMP3, have a collinear relationship with two or more LEA orthologous in *Arabidopsis thaliana* and *Oryza sativa*. In particular, GmSMP1 has a collinear relationship with two LEA members in the other four species (Table S2). These results suggest that these genes may play important roles in the evolution of the *GmLEA* gene family. 

Synonymous (Ks) and nonsynonymous (Ka) values were further calculated to explore the selective pressure on duplicated *GmLEA* genes. In general, a Ka/Ks ratio greater than 1 indicates positive selection, a ratio less than 1 indicates functional constraint, and a Ka/Ks ratio equal to 1 indicates neutral selection [27]. The orthologous *GmLEA* gene pairs were used to estimate Ka, Ks, and Ka/Ks (Table S3). The results revealed that the Ka/Ks ratios of most *GmLEA* genes were greater than 0.1 but less than 1.0, with most ranging from 0.1 to 0.6. The lowest Ka/Ks ratio was only 0.0532, and the highest was 1.264. The Ka/Ks ratio of the LEA3_4 and LEA3_7 genes exhibited relatively high Ka/Ks ratios (greater than 1), indicating that they might preferentially conserve the function and structure under positive selective pressure.

### 2.4. Cis-Elements Analysis in Promoters of GmLEA Genes

**Figure 5:**
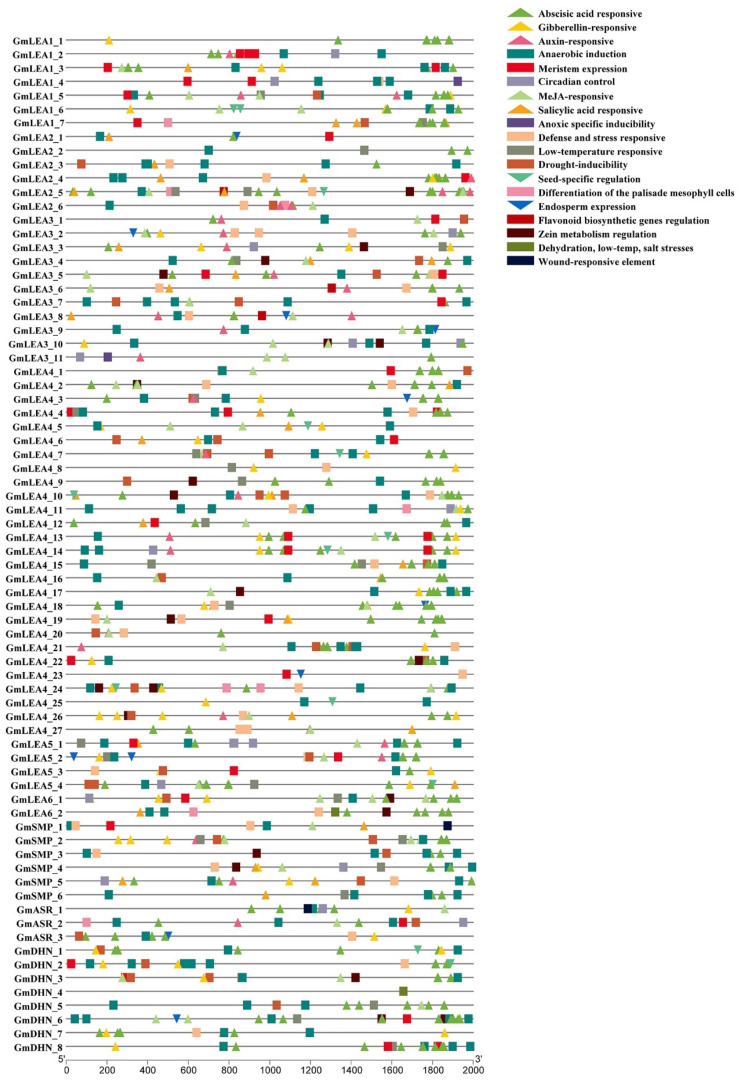
Cis-acting elements in the promoter region of the soybean *LEA* genes. The 2000 bp promoter region upstream of the gene was analyzed. Different colored boxes represent different cis-acting elements.

A total of 2000-bp promoter sequences from each *GmLEA* gene were extracted and used for cis-element prediction (Figure 5). The hormone-related cis-regulatory elements, including methyl jasmonate (MeJA)-responsive elements, gibberellin-responsive elements, abscisic acid (ABA) response elements, auxin response elements, and salicylic acid- responsive elements, were enriched. ABA-responsive elements were found in many genes. Moreover, stress-related cis-regulatory elements, including anaerobic induction elements, low-temperature-responsive elements, defense- and stress-responsive elements, drought-inducibility elements, and anoxic-specific inducibility elements were identified. These elements were involved in plant responses to dehydration, low temperature, salt stress, and flooding stresses. In addition, the promoters of 22 *GmLEA* genes contained seed-specific regulation elements or endosperm expression cis-regulatory elements, indicating a strong relationship between the GmLEA family and seed expression patterns.

### 2.5. Prediction of Regulatory Factors and miRNA Targets on GmLEA Transcripts

We used the promoter sequence to predict the potential regulatory interactions between transcription factors (TFs) and *GmLEA* genes. A total of 340 TFs were found to be involved in the expression regulation of 74 GmLEAs (Table S4). Among these TFs, bHLH and ERF contained a higher proportion of binding sites. Furthermore, to explore more information on *GmLEA* gene functions, we conducted the prediction of miRNAs targets on LEA transcripts (mRNA) using psRNATarget. A total of 56 *GmLEA* genes were found to be targeted by 119 miRNAs, representing 76% of all *GmLEA* genes (Table S5). The highest levels of targeting were detected on the following genes with more than 10 miRNAs: GmDHN_6 (10 miRNAs), GmASR_2 (12 miRNAs), GmLEA4_18 (14 miRNAs), GmLEA4_6 (15 miRNAs), GmLEA4_2 (16 miRNAs), GmLEA4_16 (16 miRNAs), GmLEA4_22 (19 miRNAs), and GmLEA4_15 (20 miRNAs) (Table S5). Several specific miRNAs had high levels of targeting to various genes such as gma-miR1535a (eight genes), gma-miR1535b (eight genes), gma-miR9742 (nine genes), and gma-miR9752 (11 genes).

### 2.6. Expression Profiles Analysis of GmLEA Genes across Tissues

**Figure 6:**
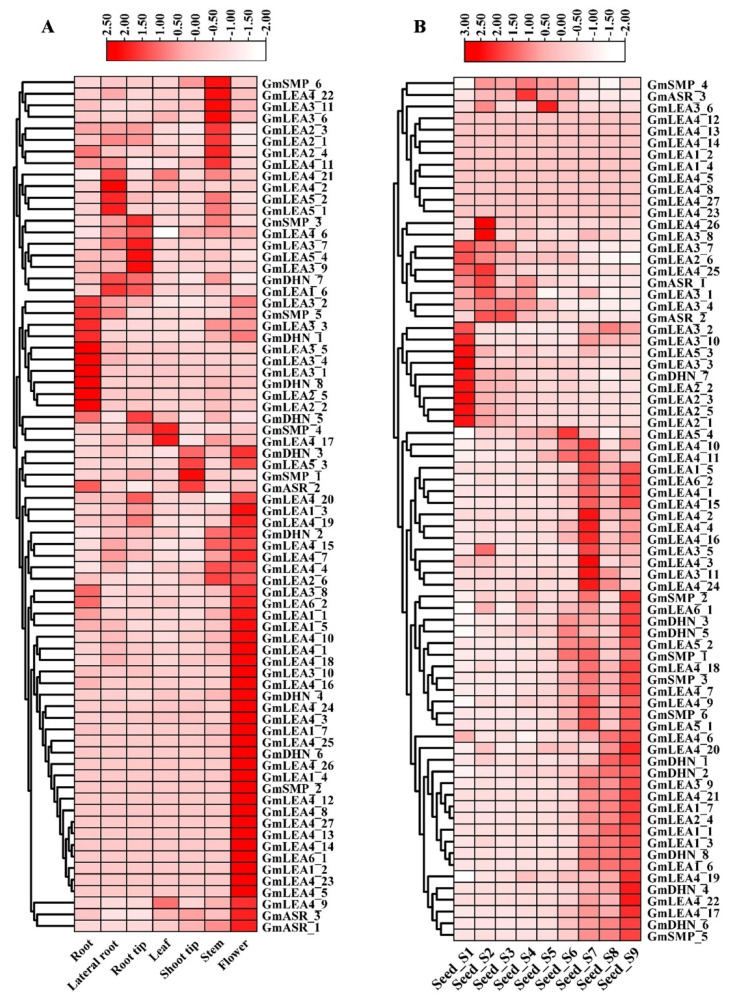
Heatmap and clustering diagram of *GmLEA* gene expression in different tissues based on normalized row scale method. (**A**) and seed stage of development (**B**). Rows represent GmLEA members, while columns show different developmental stages and tissues. Blocks with the intensity of colors indicate decreased (white) or increased (red) transcript accumulation.

The availability of published transcriptome data facilitates the study of the basic biology of soybean. The tissue expression data were obtained for *GmLEA* genes in the root, lateral root, root tip, leaf, shoot tip, stem, and flower tissues. Among the 74 *GmLEA* genes, the majority were expressed at low or undetectable levels in the analyzed tissues, with FPKM values < 10. However, the GmASR, GmLEA2, and GmLEA3 subfamily were expressed at higher levels in most of the analyzed tissues (Table S6). In addition, approximately 90% of *GmLEA* genes showed tissue-specific expression patterns (Figure 6A). For example, *GmSMP_6*, *GmLEA4_22*, *GmLEA4_11*, *GmLEA3_6*, *GmLEA3_11*, *GmLEA2_1*, *GmLEA2_3*, and *GmLEA2_4* were mainly expressed in stem tissue. *GmSMP_4* and *GmLEA4_17* was highly expressed in leaves. Moreover, half of the *GmLEA* genes were specifically and highly expressed in flower tissue compared to other tissues. 

Due to the presence of the endosperm expression cis-element in the promoter regions of most *GmLEA* genes, we further compared the expression patterns of all *GmLEA* genes at different seed stages. As illustrated in Figure 6B, nine *GmLEA* genes were not expressed, 21 *GmLEA* genes were highly expressed in the early seed development stages (S1–S6), and the remaining 44 *GmLEA* genes were highly expressed in the late seed development stages (S7–S9). In addition, the promoter regions of *GmDHN_1* and *GmDHN_2* genes contained seed-specific regulation cis-elements and were predominantly expressed in the late stages of seed development. These results suggest that GmLEA plays a crucial role in seed maturation and dehydration processes.

### 2.7. Expression of GmLEA Genes in Response to Abiotic Stress

**Figure 7:**
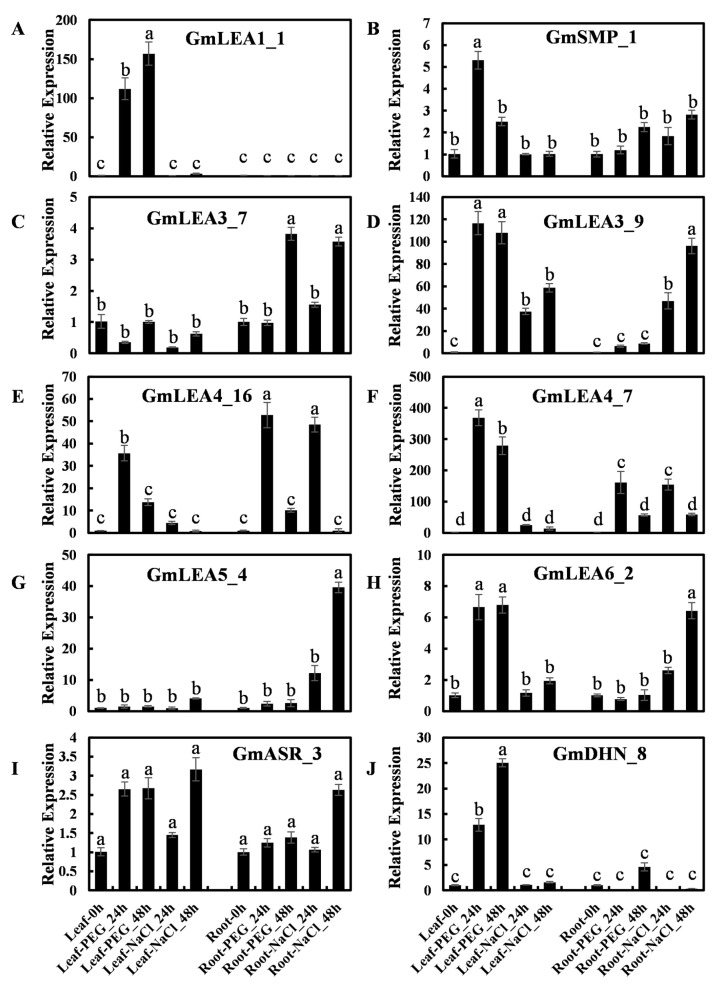
qRT-PCR expression patterns of ten *GmLEA* genes under salt and PEG6000 treatments. The time points represented by x-axis and the scale of relative expression shown by y-axis. Within the figure, columns with different letters are significantly different (from a Tukey–Kramer HSD P, 0.01). (**A**–**J**) The relative expression levels of ten *GmLEA* genes revealed by qPCR.

To further investigate the expression pattern of *GmLEA* genes in response to abiotic stress, qRT-PCR was performed on 10 *GmLEA* genes under salt- or water-deficit stresses. The results indicated that the accumulation of *GmLEA* genes was associated with different tissues and treatments, and the expression pattern also differed within each subfamily (Figure 7). For example, the expression of *GmLEA1_1* increased in leaves under PEG treatment, while no significant changes were found in root tissues or under salt treatment (Figure 7A). *GmLEA5_4* was highly induced by salt treatment in root tissue whereas no significant change was observed in leaf tissue or under PEG treatment (Figure 7G). Interestingly, some phylogenetically related gene pairs exhibited different expression patterns. For example, *GmLEA3_9* was highly induced by PEG and salt treatment in leaves and by salt in roots (Figure 7D). The expression levels of *GmLEA3_7* decreased in response to 24 h PEG and salt treatment in leaves but increased in response to 48 h PEG and salt treatment in roots (Figure 7C). These results suggest that even though these genes are phylogenetically related, they may be involved in the different biological pathways. In addition, the *GmASR_3* transcript was higher in flowers than other tissues and was not involved in the PEG and salt response (Figure 7I). The transcript level of *GmDHN_8* was highly induced via PEG treatment in leaves but not in roots, even though it has the predominant expression pattern in roots (Figure 7J). 

### 2.8. Co-Expression-Based Gene Network Analysis of GmLEA Genes

**Figure 8:**
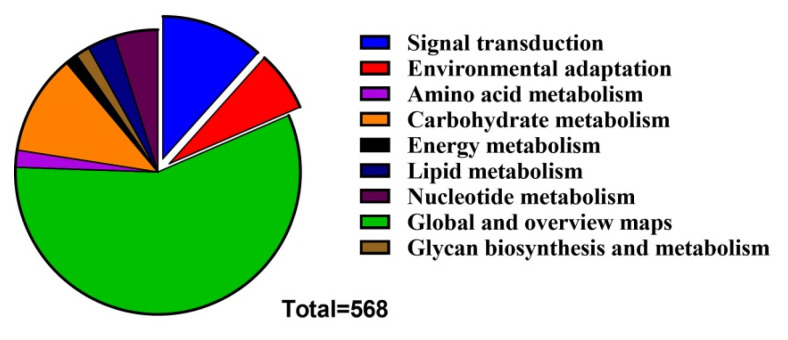
Pie chart representing the enriched KEGG pathways found within genes that co-expressed with all *GmLEA* genes in *Glycine max*.

All *GmLEA* genes were selected for the co-expression-based gene network analysis. Co-expressed genes with Spearman correlation coefficients were selected as relevant genes from the RNA-Seq data. A total of 3196 genes were selected based on a *p*-value < 0.05. Finally, 568 genes with significant enrichment in the different pathways were identified. The Kyoto Encyclopedia of Genes and Genomes (KEGG) enrichment analysis revealed that the metabolism-related pathways were enriched in these co-expressed genes, including amino acid metabolism, carbohydrate metabolism, energy metabolism, global and overview maps, glycan biosynthesis and metabolism, lipid metabolism, and nucleotide metabolism (Figure 8). For signal transduction, it mainly involved the signal transduction pathways of plant hormones. Environmental adaptation mainly involved the plant-pathogen interaction pathways. Those results suggest that soybean LEA proteins may exert regulatory effects on those metabolic pathways. 

### 2.9. Overexpression of GnLEA4_19 Improved the Drought Tolerance in Arabidopsis and Soybean

**Figure 9:**
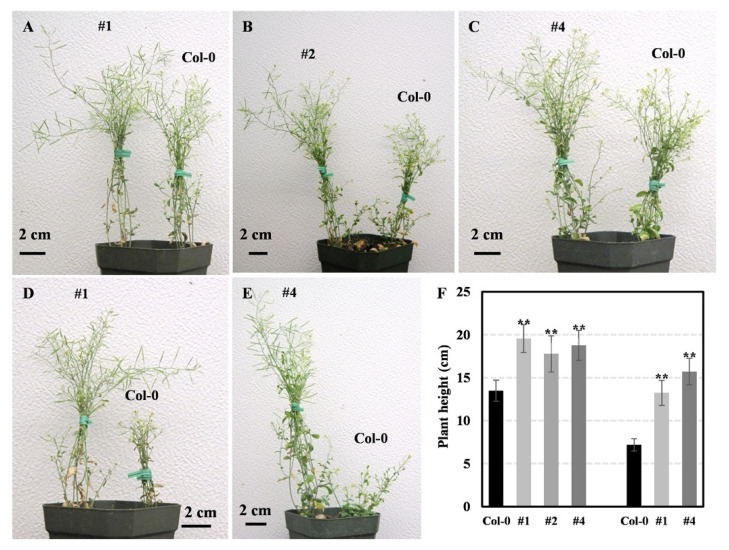
Overexpressed GmLEA4_19 increased plant height than wild type under drought condition. (**A**–**C**) Under mild drought condition; (**D**,**E**) under serious drought condition; and (**F**) plant height was measured under both mild drought condition and serious drought condition. Means and standard deviations were obtained from three biological replicates. Asterisks represent statistically significant differences between wild-type and transgenic lines under the same treatment. **, *p* < 0.05.

To explore the functions of GmLEA, three independent *Arabidopsis* transgenic lines (ABRE3:GmLEA4_19_#1, ABRE3:GmLEA4_19_#2, and ABRE3:GmLEA4_19_#4) were generated and used to conduct drought assays. Under a mild drought condition, the plant height in transgenic plants was significantly higher than wild plants (Figure 9A–C). Under a serious drought condition, the average height of transgenic plants is twice that of wild-type plants (Figure 9D–F). Moreover, the seed setting rate of the wild-type significantly decreased, but some pods of the transgenic plants were still able to grow normally (Figure 9D,E).

**Figure 10:**
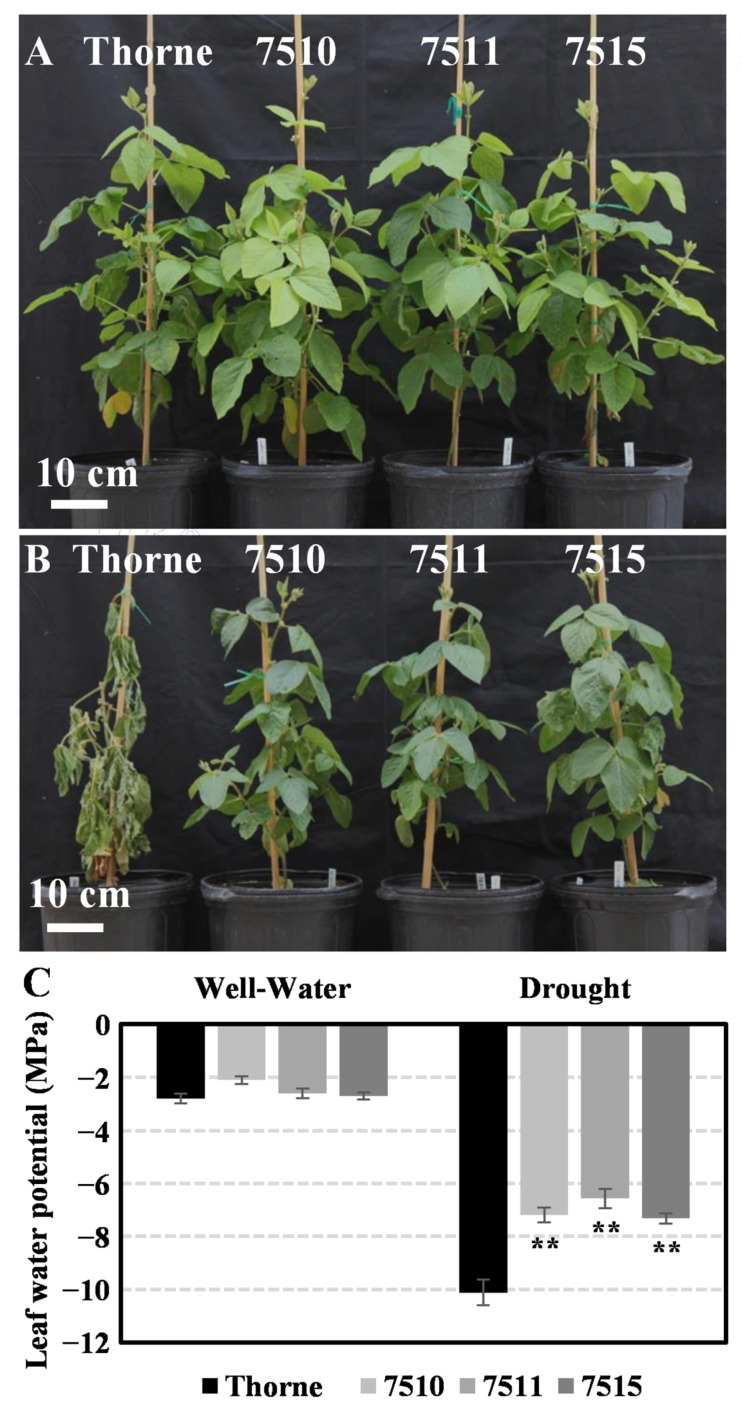
Overexpressed *GmLEA4_19* transgenic soybean showed drought tolerance phenotype. (**A**) Transgenic plants growing in the greenhouse under well-watered condition; (**B**) transgenic plants growing in the greenhouse after withholding water for 21 days; and (**C**) leaf water potential was determined under well water and drought conditions. Means and standard deviations were obtained from three biological replicates. Asterisks represent statistically significant differences between wild-type and transgenic lines under the same treatment. **, *p* < 0.05.

To further determine the role of GmLEA4_19 in soybean, three independent overexpressing transgenic soybean lines (7510, 7511, and 7515) were generated and used to conduct drought assays. Under a well-water condition, the leaf water potential in transgenic plants had no significant difference compared with the non-transgenic control plants (Figure 10A). After withholding water for 21 days, in comparison to the controls, ABRE3:GmLEA4_19 transgenic soybean lines showed slower wilting than the controls (Figure 10B). Moreover, we also found that the leaf water potential of the transgenic plants was significantly less than the controls under the drought condition (Figure 10C). These results indicate that overexpressing *GmLEA4_19* transgenic plants were more tolerant to drought.

## 3. Discussion

The *LEA* gene family plays a vital role in multiple physiological processes in response to abiotic stress in plants, such as *Arabidopsis*, maize, Cassava, and *Larix kaempferi*. However, there is limited information available on the regulation and structure of these genes in *Glycine max*. In this study, we examined 74 GmLEAs from the soybean genome, which is an expanded number compared to Arabidopsis, maize, Cassava, Moso Bamboo, and *Cleistogenes songorica*, but it also fewer than poplar, sugarcane, *brassica napus*, wheat, and upland cotton. The number of LEA family members in soybean may be correlated with genome size and suggests the key role this gene family played in soybean growth and development. 

Gene duplication is a key feature of gene family expansion and can occur using three models: segmental duplication, tandem duplication, and whole-genome duplication [28,29]. Investigating the gene duplication mechanism of *GmLEA* genes can help us understand the diversification of gene function. Segmental duplication events were found in 57 pairs of paralogous *GmLEA* genes. Most of these paralogous gene pairs showed similar exon–intron organization, except for the GmLEA4 subfamily, which had different exon–intron organizations. According to the descriptions of Holub [30], a chromosomal region within 200 kb containing two or more genes is considered a tandem duplication event. In the GmLEA family, six tandem duplication events were identified. These results confirm that the total number of *GmLEA* genes expanded via both tandem and segmental duplication. Additionally, the syntenic analysis revealed that *GmLEA* genes had higher homology with the *LEA* genes from dicotyledons and lower homology with monocotyledons. This finding suggests that the GmLEA family may have evolved separately from dicotyledons and monocotyledons. 

Low hydrophobicity and a large net charge are characteristics of LEA proteins that allow them to be either completely or partially disordered. Therefore, these proteins could form flexible structural elements to binding water that help protects the plants from desiccation or dehydration [31,32,33]. While no same conserved domains have been identified among the different subfamilies of LEA proteins, most LEA proteins share the same physical and chemical characteristics with a glycine ratio higher than 6% and hydrophilicity greater than 1 [34,35,36]. The same protein characteristics were also found in soybean LEA proteins in this study. Therefore, the high glycine content in GmLEA proteins may contribute to their hydrophilic nature and ability to enhance the stability of proteins and membranes, which could help protect cells from desiccation or dehydration during periods of environmental stress or different seed development stages. 

Earlier studies of LEA family genes in other plants emphasized their role in response to abiotic stress, especially drought. The overexpression of *LkDHNs* (a dehydrin gene in *Larix kaempferi*) improves the osmotic tolerance of tobacco protoplasts and enhanced the survival rate in yeast under heavy osmotic stress [37]. The reduction in the *CaDIL1* (a pepper LEA protein) transcripts in pepper exhibited reduced drought tolerance and ABA sensitivity [38]. It has been reported that there is a high correlation between the LEA accumulation and the water deficit, reinforcing their functional relevance under these detrimental conditions [39]. In the current study, the expression patterns of 10 *GmLEA* genes in response to the NaCl and PEG6000 treatments suggested that these genes had essential roles in the abiotic stress responses of soybean. GmLEA1_1 was significantly induced by PEG6000 and there are many ABA responsive elements in the promoter region of this gene, which means that the fast-induced expression on the exogenous PEG treatment may by correlated with the ABA hormone. GmLEA5_4 was induced by NaCl in root tissue. The staging of seed development is based on the fresh weight/color system described by Meinke et al. [40] and Jones and Vodkin [41]. Here, we found over half the number of *GmLEA* genes were highly and specifically expressed in mature yellow seeds (S7) and fully mature, yellow, dehydrating seeds (S8); the seeds are quiescent, yellow/tan-colored, and fully dehydrated (S9). Therefore, we could propose that those GmLEA proteins play important roles in the seed maturation process, which may help preserve the cellular structures and nutrients within the seed during desiccation. 

The LEA overexpressed plants maintain higher superoxide dismutase, catalase (CAT), and ascorbate-peroxidase activities, and accumulated more proline and less malondialdehyde (MDA) compared with the wild-type plants under abiotic stress conditions [42,43]. However, the *MsLEA4-4* overexpression in *Arabidopsis* had a high level of soluble sugar, and there was activity of various antioxidant enzymes while the levels of proline and malondialdehyde were significantly reduced [19]. The overexpression of the *SiDHN* gene has been shown to enhance the cold and drought tolerance of transgenic tomato plants. This is achieved by preventing cell membrane damage, protecting chloroplasts, and increasing the plant’s ability to scavenge reactive oxygen species [44]. Additionally, an increasing number of studies have found that LEA may be involved in more regulatory mechanisms. For example, the mutant of AtLEA13 and AtAtLEA30 were found to be more sensitive to drought stress due to their increased transpiration and increased stomatal density [45]. The overexpression of the *OsLEA1a* gene in rice could protect plants from various abiotic stresses by preventing cell membrane damage and increasing the plant’s ability to scavenge reactive oxygen species [37]. The GWAS analysis revealed LEA3 loci play a significant role in grain mold resistance in sorghum [46]. It was reported that NaCl treatment enhanced the signal of LkDHNs in the nucleus, indicating that LkDHNs may play roles in the plant cell nucleus under stress [47]. Arabidopsis LEA5 regulates organellar translation to enhance cellular respiration relative to photosynthesis when coping with stress [48]. The overexpression of *TaLEA2-1* in wheat “1718” led to greater height, stronger roots, and higher catalase activity than in wild type seedlings [49]. The above results indicate that the function of the GmLEA protein is complex. LEA proteins may function as a hub to cross talk with various molecules and pathways. In our study, the role of GmLEA4_19 should be further explored to elucidate the molecular mechanism under osmotic stress. 

## 4. Materials and Methods

### 4.1. Identification of GmLEA Genes in Glycine Max

Soybean-predicted proteins were retrieved from the Phytozome database (https://phytozome-next.jgi.doe.gov/v13, accessed on 12 April 2022) [50]. Putative GmLEA proteins were initially identified as candidates annotated as *LEA* genes. Typical Pfam protein domains, including PF0477(Group 1, LEA_5), PF00257(Group 2, dehydrin, DHN), PF02987(Group 3, LEA_4), PF03760(Group 4, LEA_1), PF04927(Group 5A, SMP), PF03242(Group 5B, LEA_3), PF03168(Group 5C, LEA_2), PF10714(Group 6, LEA_6), and PF02496(Group 7, ASR) were then used as queries to identify the *GmLEA* genes. The conserved domains in the LEA protein sequences identified in *G*. *max* were further examined using the Pfam 35.0 (https://pfam.xfam.org/, accessed on 28 September 2023) and HMMER tool (https://www.ebi.ac.uk/Tools/hmmer/, accessed on 28 September 2023). Protein sequences without the LEA conserved domains were removed. *GmLEA* genes were finally named according to their domain types and positions on the chromosomes.

### 4.2. Analysis of GmLEA Protein Properties

The molecular weight (MW), theoretical isoelectric point (pI), instability index, and grand average of hydrophobicity (GRAVY score) of GmLEA were predicted using the ExPASy website (http://web.expasy.org/protparam/ (accessed on 28 September 2023) [51]). Protein Prowler Subcellular Localization Predictor version 1.2 (http://bioinf.scmb.uq.edu.au/pprowler_webapp_1-2/, accessed on 28 September 2023) [52] and Plant-mPLoc (http://www.csbio.sjtu.edu.cn/bioinf/plant-multi/#, accessed on 28 September 2023) servers [53] were used to predict the subcellular locations of all GmLEA proteins.

### 4.3. Phylogenetic and Conserved Motifs Analysis of GmLEA Proteins

GmLEA proteins were aligned using MAFFT version 7 software [54] to generate a FASTA alignment file. A neighbor-joining (NJ) tree was constructed using MEGA 11 with 1000 bootstrap replications. The phylogenic tree was displayed using iTOL v5 [55]. The amino acid sequences of GmLEAs were analyzed using the Multiple Expectation Maximization for Motif elicitation (MEME) tool (http://meme-suite.org/index.html, accessed on 28 September 2023) to identify the conserved domains and motifs in each group. The maximum number of motifs was set to 3, with a minimum width of 6 and a maximum width of 50 amino acids residues, and an e-value < 1 × 10^−8^. 

### 4.4. Chromosomal Location, Gene Structure, and Gene Duplication of GmLEA Genes

The chromosomal location of *GmLEA* genes was retrieved from the Glycine max genome data. The exon–intron structures of *GmLEA* genes were analyzed by aligning the coding sequences with the corresponding genomic sequences and visualized using TBtools software (v2.008) [56]. Duplicate events of *GmLEA* genes were determined using MCscan pairs [57]. In addition, Dual Synteny Plotter was used to analyze the collinearity between the GmLEA and homologous genes from four other species (Vigna unguiculata, Sorghum bicolor, Arabidopsis, and Oryza sativa), which was visualized using TBtools software [56]. We used the Ka/Ks Calculator (NG) to obtain the ratio of non-synonymous substitution and synonymous substitution (Ka/Ks) for the duplication gene pairs. We also applied the methods of Koch [58] to calculate the divergence time of each gene pair.

### 4.5. Regulatory Networks Analysis

For the cis-acting elements analysis, the 2000 bp DNA sequence upstream from the start codon of all the *GmLEA* genes were retrieved from the genome database of G.max and were queried via the PlantCARE database (http://bioinformatics.psb.ugent.be/webtools/plantcare/html/ (accessed on 28 September 2023) [59]). The abundant motifs were visualized by use of the TBtools software [56]. The binding sites of the GmLEA gene’s promoter were predicted using the Plant Transcriptional Regulatory Map (http://plantregmap.gao-lab.org/regulation_prediction_result.php (accessed on 28 September 2023) [60]) with a threshold (for binding site prediction) of *p*-value ≤ 1 × 10^−5^. 

For the gene co-expression analysis, all *GmLEA* genes were used for the co-expression-based gene network analysis. We used the Spearman correlation coefficients to select relevant genes from the RNA-Seq data. Gene selection was based on *p*-value < 0.05. 

For the prediction of the miRNA-targeted GmLEA genes, the miRNA database (Glycine max, 639 published miRNA) was selected and all GmLEA genes targeted via miRNAs were predicted by searching the coding sequences by using the psRNATarget server with default parameters (Schema V2) (http://plantgrn.noble.org/psRNATarget/?function=2, accessed on 28 September 2023) [61].

### 4.6. Tissue Expression Pattern Analysis Based on RNA Sequencing Data

To analyze the tissue expression patterns of *GmLEA* genes, the expression pattern was downloaded from the JGI Plant Gene Atlas. Heatmaps were generated using TBtools to display the expression profiles of *GmLEA* genes [56]. The fragments per kilobase of exon model per million mapped read (FPKM) values of *GmLEA* genes were visualized. Flower tissue was collected from the opened flowers that had grown in the field in the flowing stage. Root, lateral root, root tip, shoot tip, leaf, and stem tissues were collected from 4-week-old plants grown on the B&D medium [62]. The seed stage is based on the weight ranges as follows: S1 < 10 mg; S2, 30–50 mg (storage cells have large central vacuoles); S3, 70–90 mg (storage protein accumulation has begun, and subdivision of the vacuole is occurring); S4, 115–150 mg; S5, 200–250 mg (filling of the storage vacuoles); S6, >300 mg (green color seeds); S7, >300 mg (yellow color seeds); S8, 200–250 mg (fully-mature, yellow color, and dehydrating seeds); and S9 < 150 mg (yellow color seeds and fully dehydrated).

### 4.7. Agrobacterium-Mediated Soybean (Glycine Max) and Arabidopsis Transformation

The gene-specific primer pair 5′-GGAGCTCATGGCATCCCATAGGCAAAGC-3′ and 5′-TCCCCGGGGTAATTTCTGCGGTTGTCTTG-3′ was designed to isolate the full-length CDS of GmLEA4_19 from soybean. The PCR product (423bp) was cloned into the Topo vector for sequencing. The positive clone was cut with SacI and SmaI to make the pRTL2-ABRC3 subcloning vector before fusion with the ABRC3 promoter. Finally, the whole cassata was cloned into the pPTN200 binary vector. For the soybean transformation, an improved Agrobacterium-mediated transformation of the soybean cotyledonary node system was performed using the elite genotype “Thorne” [63]. The transgenic soybean plants were screened using the leaf paint (100 mg/L glufosinate, Sigma, St. Louis, MO, USA) analysis and the transgenic Arabidopsis were screened using 10 μg/mL of basta. The abiotic-resistance seedlings were verified via PCR analysis using specific primers. The homozygous lines used for subsequent phenotype studies.

### 4.8. Plant Materials Growth Conditions and Treatments

Soybean Williams 82 seeds were germinated on a Petri dish lined with moist filter paper. Seedlings were then transferred to a growth chamber and grown in a half-strength MS solution under a 10 h photoperiod at 25 °C during the day and 22 °C at night. At the vegetative 1 stage, the plants were transferred to a half MS solution containing either 15% PEG6000 (*Ψ*s −0.388 MPa) [64] or 150 mM of NaCl for 24 and 48 h, respectively. The roots and first trifoliolate leaves from five plants were harvested for the *GmLEA* gene expression analysis. Samples were immediately frozen in liquid nitrogen after harvest and stored at −80 °C for total RNA isolation. 

For the analysis of transgenic soybean phenotypes, the transgenic and control soybean seeds were planted in soil-filled pots. The plants were grown until they reached the V4-V5 stage (around 5 weeks), after which they were withholding water for 21 days. The greenhouse temperature was maintained at 24~26 °C during both day and night. The shade was always kept open, and the HID lights were set to be on from 5 a.m. and 7 p.m. 

For the analysis of transgenic Arabidopsis phenotypes, the wild-type and three independent transgenic lines were sown on a half MS medium for 10 days at 23 °C under a 16 h light/8 h dark cycle. Both wild-type and transgenic seedlings were then transferred into the same containers filled with soil. The seedlings were watered regularly for 2 weeks. For the mild drought treatment, the soil-relative water content was maintained at 55% and kept for 4 weeks. For the serious drought treatment, the soil-relative water content was maintained at less than 40% and kept for 4 weeks.

### 4.9. RNA Isolation, cDNA Synthesis, and qRT-PCR

The transcript abundance of several *GmLEA* genes was investigated using qRT-PCR. Total RNA was extracted from the roots and leaves of the G. max seedling under the stress treatment and the unstressed control using an RNApure Plant Kit (DNase I) (CWBIO, Cat: # CW0559, Taizhou, China) according to the manufacturer’s instructions. Approximately 2 μg of total RNA was reverse transcribed into cDNA in a 20 μL reaction volume using the HiScript 1st Strand cDNA Synthesis Kit (Vazyme, Cat: # R111-01, Nanjing, China) following the supplier’s instructions. Quantitative RT-PCR was performed using the Bio-Rad CFX ConnectTM Optics Module Real-Time PCR System (Bio-Rad, Ontario, CA, USA) and iTaq Universal SYBR Green Supermix (Bio-Rad, Cat: #1725122, Ontario, CA, USA). The constitutive Gmactin11 gene, with forward “ATCTTGACTGAGCGTGGTTATTCC” and reverse sequence “GCTGGTCCTGGCTGTCTCC” was used as a reference gene and specific LEA genes primers were used for qRT-PCR validation. The relative gene expression data obtained via qRT-PCR were normalized to the expression of the GmActin gene. The 2^-ΔΔCt^ method was used to calculate the relative expression of GmLEA genes. Each sample has three replicates, and three biological experiments were performed. The primers used for qRT-PCR are listed in Table S7.

## 5. Conclusions

This study conducted a comprehensive analysis of the GmLEA family in soybean. A total of 74 *GmLEA* genes were identified and classified into nine subfamilies. The evolutionary characteristics and expression patterns of these genes in different soybean tissues provide valuable clues about the evolution of LEAs. The expression patterns of several GmLEA in response to drought and salt stress may help to further understand the functions of GmLEA members under the stress condition. Our studies suggest that GmLEA4_19 may function in regulating plant height and drought tolerance. Taken together, these results provided insight for better understanding the biological roles of the *LEA* genes in soybean. 

## Figures and Tables

**Table 1:** *LEA* genes in soybean genome and their protein sequence characteristics.

Gene Name	Gene ID	PFAM ID	PFAM	Motif #	# of Amino Acids	Molecular Weight	Theoretical pI	Instability Index
GmLEA1_1	Glyma.03G144400	PF03760	LEA_1	1	152	15,581.23	9.66	21.04
GmLEA1_2	Glyma.04G128500	PF03760	LEA_1	1	101	11,069.41	6.85	24.22
GmLEA1_3	Glyma.05G112000	PF03760	LEA_1	1	131	14,628.44	9.07	48.27
GmLEA1_4	Glyma.06G310300	PF03760	LEA_1	1	101	11,006.42	6.92	9.7
GmLEA1_5	Glyma.09G112100	PF03760	LEA_1	1	222	23,228.42	6.17	30.92
GmLEA1_6	Glyma.17G155000	PF03760	LEA_1	1	133	14,680.46	9.27	49.32
GmLEA1_7	Glyma.19G147200	PF03760	LEA_1	1	173	17,606.29	9.58	13.11
GmLEA2_1	Glyma.02G277300	PF03168	LEA_2	1	321	35,813.72	4.92	25.83
GmLEA2_2	Glyma.09G254302	PF03168	LEA_2	1	152	16,551.14	4.85	17.14
GmLEA2_3	Glyma.14G037300	PF03168	LEA_2	1	381	42,615.88	4.75	24.75
GmLEA2_4	Glyma.16G031300	PF03168	LEA_2	1	152	16,688.34	5.16	21.68
GmLEA2_5	Glyma.18G238700	PF03168	LEA_2	1	176	18,933.17	5.83	17.91
GmLEA2_6	Glyma.20G044800	PF03168	LEA_2	1	314	34,616.47	4.79	16.24
GmLEA3_1	Glyma.02G017100	PF03242	LEA_3	1	98	10,299.65	9.7	33.61
GmLEA3_2	Glyma.03G215000	PF03242	LEA_3	1	98	10,513.96	9.43	51.92
GmLEA3_3	Glyma.03G253200	PF03242	LEA_3	1	90	9770.06	9.85	38.99
GmLEA3_4	Glyma.09G043400	PF03242	LEA_3	1	97	10,388.76	10.08	63.7
GmLEA3_5	Glyma.10G017600	PF03242	LEA_3	1	95	10,015.34	9.57	30.6
GmLEA3_6	Glyma.10G259200	PF03242	LEA_3	1	101	10,767.06	9.06	34.18
GmLEA3_7	Glyma.15G149600	PF03242	LEA_3	1	100	10,655.93	10.41	74.69
GmLEA3_8	Glyma.16G013200	PF03242	LEA_3	1	93	10,767.44	9.55	47.41
GmLEA3_9	Glyma.17G027400	PF03242	LEA_3	1	113	12,282.97	10.09	56.12
GmLEA3_10	Glyma.19G211600	PF03242	LEA_3	1	98	10,682.2	8.93	45.61
GmLEA3_11	Glyma.20G131700	PF03242	LEA_3	1	101	10,982.39	9.51	28.55
GmLEA4_1	Glyma.03G189200	PF02987	LEA_4	3	316	35,342.05	5.96	32.87
GmLEA4_2	Glyma.06G283900				1069	1069	8.55	42.09
GmLEA4_3	Glyma.07G032400		LEA_4	0.00081	136	14,836.22	8.73	29.36
GmLEA4_4	Glyma.08G239400	PF13664			383	43,024.39	8.36	38.31
GmLEA4_5	Glyma.09G252700				155	16,701.6	9.65	30.8
GmLEA4_6	Glyma.10G014200				208	23,104.15	9.65	35.56
GmLEA4_7	Glyma.10G064400	PF02987	LEA_4	6	449	48,795.56	6.12	25.23
GmLEA4_8	Glyma.10G130600				88	9250.01	5.68	31.37
GmLEA4_9	Glyma.11G068900				296	32,056.4	5.63	30.6
GmLEA4_10	Glyma.12G001600				341	38,257.19	9.43	39.37
GmLEA4_11	Glyma.12G209500				540	57,273.13	5.43	32.19
GmLEA4_12	Glyma.13G050000				65	6712.32	6.06	42.02
GmLEA4_13	Glyma.13G050051				65	6668.26	8.1	26.48
GmLEA4_14	Glyma.13G050100				65	6682.29	8.1	30.1
GmLEA4_15	Glyma.13G119400				473	50,982.23	6.65	30.84
GmLEA4_16	Glyma.13G149000	PF02987	LEA_4	7	463	50,643.82	6.33	30.29
GmLEA4_17	Glyma.13G237700				233	25,630.03	5.5	32.31
GmLEA4_18	Glyma.13G291800				643	67,977.14	6.18	29.76
GmLEA4_19	Glyma.13G363300				140	15,097.38	8.95	37.62
GmLEA4_20	Glyma.15G010500				101	11,100.1	6.73	22.79
GmLEA4_21	Glyma.15G075700				145	16,464.96	5.03	25.52
GmLEA4_22	Glyma.17G040800				458	49,399.65	7.08	29.87
GmLEA4_23	Glyma.18G240000				159	16,983.91	9.45	17.5
GmLEA4_24	Glyma.18G278700				63	6593.26	9.05	24.83
GmLEA4_25	Glyma.18G279300				66	6808.39	7.92	28.75
GmLEA4_26	Glyma.19G040000				62	6450.99	4.72	40.22
GmLEA4_27	Glyma.20G081400				88	9258.04	6.71	29.55
GmLEA5_1	Glyma.01G119600	PF00477	LEA_5	2	101	11,141.06	6.31	42.97
GmLEA5_2	Glyma.03G056000	PF00477	LEA_5	2	105	11,505.35	5.53	44.21
GmLEA5_3	Glyma.07G152400	PF00477	LEA_5	1	83	9369.31	6.59	43.53
GmLEA5_4	Glyma.18G203500	PF00477	LEA_5	1	112	12,246.34	5.33	46.21
GmLEA6_1	Glyma.05G103100	PF10714	LEA_6	1	105	11,440.67	9.05	52.21
GmLEA6_2	Glyma.17G164200	PF10714	LEA_6	1	95	10,060.89	4.91	54.59
GmASR_1	Glyma.10G224300	PF02496	ABA_WDS	1	213	23,058.3	5.7	38.31
GmASR_2	Glyma.16G166600	PF02496	ABA_WDS	1	111	12,625.9	6.35	30.1
GmASR_3	Glyma.20G167500	PF02496	ABA_WDS	1	238	25,353.7	5.58	37.27
GmDHN_1	Glyma.04G009400	PF00257	Dehydrin	1	214	24,164.6	5.53	50
GmDHN_2	Glyma.04G009900	PF00257	Dehydrin	1	166	17,319.92	9.22	34.1
GmDHN_3	Glyma.08G048900	PF00257	Dehydrin	1	91	9917.88	6.64	30.22
GmDHN_4	Glyma.12G235800	PF00257	Dehydrin	1	135	14,870.27	5.54	32.59
GmDHN_5	Glyma.13G201300	PF00257	Dehydrin	1	139	15,133.43	5.52	38.07
GmDHN_6	Glyma.09G185500	PF00257	Dehydrin	1	253	26,630.01	6.29	8.95
GmDHN_7	Glyma.06G009350	PF00257	Dehydrin	1	153	17,521.21	5.56	46.52
GmDHN_8	Glyma.07G090400	PF00257	Dehydrin	1	243	25,658.97	6.02	5.45
GmSMP_1	Glyma.10G027600	PF04927	SMP	3	262	27,455.59	5.16	26.15
GmSMP_2	Glyma.10G159400	PF04927	SMP	3	284	29,451.95	6.48	29.05
GmSMP_3	Glyma.10G247500	PF04927	SMP	3	256	26,239	4.75	37.14
GmSMP_4	Glyma.11G158394	PF04927	SMP	2	179	18,242.1	4.27	26.54
GmSMP_5	Glyma.20G147500	PF04927	SMP	1	81	8806.94	6.13	77.43
GmSMP_6	Glyma.20G147600	PF04927	SMP	3	256	26,058.06	4.9	34.86

## Data Availability

The datasets used and/or analyzed in this study are available on reasonable request from the corresponding author.

## Supplementary Materials

The following supporting information can be downloaded at: https://www.mdpi.com/article/10.3390/ijms241914834/s1.
